# Eosinophilic Fasciitis with Isolated Hand Involvement: A Diagnostic Challenge

**DOI:** 10.5152/ArchRheumatol.2025.11173

**Published:** 2025-06-23

**Authors:** Ping Wang, Mingwen Guo

**Affiliations:** Department of Internal Medicine, Qionglai Medical Center Hospital, Qionglai, China

Dear Editor,

We present this case to highlight an atypical clinical manifestation of eosinophilic fasciitis (EF), which predominantly involved the hands without systemic features—a scenario rarely reported in the literature. This case underscores the diagnostic challenges posed by localized EF and emphasizes the importance of early recognition to prevent irreversible fibrosis.

## Eosinophilic Fasciitis with Isolated Hand Involvement: A Diagnostic Challenge

**Figure 1.**
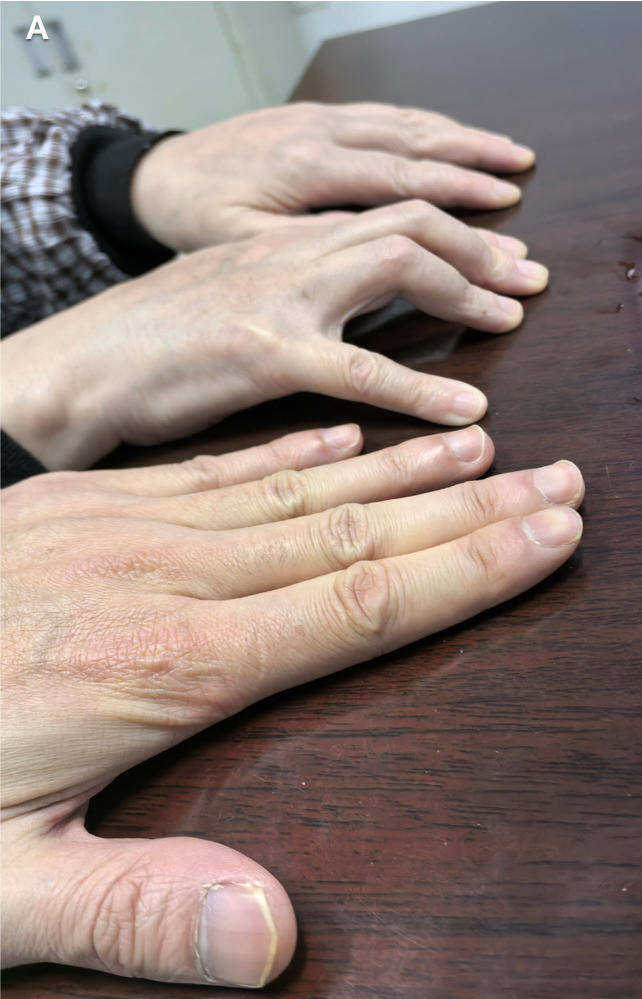
Physical examination showing indurated edema and flexion contractures of the hand (bottom panel: normal palmar surface for comparison)

**Figure 2.**
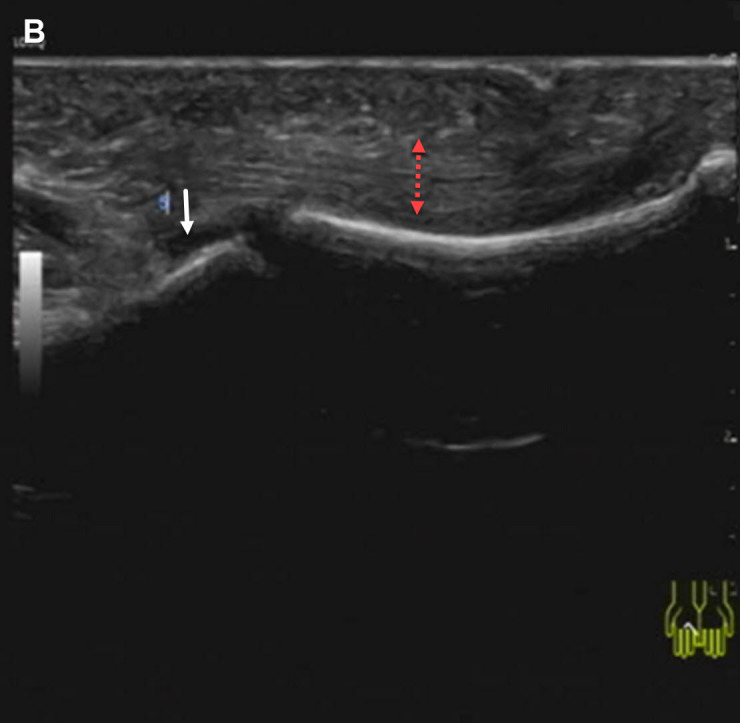
Ultrasound findings: Marked fascial thickening (5.2 mm) and mild joint effusion (arrows)

A 48-year-old woman presented with a 6-month history of progressive bilateral hand swelling, stiffness, and impaired finger extension. Physical examination revealed indurated edema and flexion contractures (Figure 1, the bottom image shows a normal palm for comparison). Laboratory tests showed peripheral eosinophilia (3.59 × 10^9^/L), elevated ESR (85 mm/h), hypergammaglobulinemia, and positive anti-SSA antibodies. Musculoskeletal ultrasound demonstrated marked fascial thickening (5.2 mm) and mild joint effusion (Figure 2, arrows).

Scleroderma was excluded by the absence of the Raynaud phenomenon, nailfold capillary changes, or visceral involvement. Dermatomyositis was ruled out due to a lack of heliotrope rash, Gottron papules, or myositis-specific autoantibodies. The diagnosis of EF was confirmed via Shulman criteria.^1^ Oral prednisone (40 mg/day) led to significant symptom resolution within 4 weeks, including restored finger mobility.

This case underscores the rarity of hand-predominant EF, which mimics scleroderma but lacks microvascular pathology. Early glucocorticoid therapy is critical to prevent irreversible fibrosis.^2^ Clinicians should consider EF in patients with sclerodactyly and eosinophilia, even without systemic features.

## Data Availability

The data that support the findings of this study are available on request from the corresponding author.
